# Supplementary material for: Chromatin accessibility landscape of articular knee cartilage reveals aberrant enhancer regulation in osteoarthritis
Source: Sci Rep. 2018 Oct 19;8:15499. doi: 10.1038/s41598-018-33779-z (PMC6195601; doi:10.1038/s41598-018-33779-z)
Supplement: Supplementary file 1 — Supplementary Figures [file 41598_2018_33779_MOESM1_ESM.pdf]

Chromatin accessibility landscape of articular knee cartilage reveals aberrant enhancer regulation in osteoarthritis

Ye Liu[1,2,3+], Jen-Chien Chang[1,2+], Chung-Chau Hon[4,5], Naoshi Fukui[6,7], Nobuho Tanaka[6], Zhenya Zhang[3], Ming Ta Michael Lee[8,9,10*], Aki Minoda[1,2*]

1. Epigenome Technology Exploration Unit, Division of Genomic Technologies, RIKEN Center for Life Science Technologies (CLST), Yokohama, Japan.
2. Epigenome Technology Exploration Unit, RIKEN Center for Integrative Medical Sciences, Yokohama, Japan.
3. Graduate School of Life and Environmental Sciences, University of Tsukuba, Tsukuba, Japan.
4. Genome Information Analysis Team, Division of Genomic Technologies, RIKEN Center for Life Science Technologies (CLST), Yokohama, Japan.
5. Genome Information Analysis Team, RIKEN Center for Integrative Medical Sciences, Yokohama, Japan.
6. Clinical Research Center, National Hospital Organization Sagamihara Hospital, Kanagawa, Japan.
7. Department of Life Sciences, Graduate School of Arts and Sciences, The University of Tokyo, Tokyo, Japan.
8. Genomic Medicine Institute, Geisinger, Danville, PA, USA.
9. Institute of Biomedical Sciences, Academia Sinica, Taipei, Taiwan, ROC.
10. Laboratory for International Alliance on Genomic Research, RIKEN Center for Integrative Medical Sciences, Yokohama, Japan.

[+] Authors contributed equally to this work

[*] Co-corresponding. Address correspondence to Aki Minoda, Epigenome Technology Exploration Unit, RIKEN Center for Integrative Medical Sciences, 1-7-22 Suehiro-cho, Tsurumi-ku, Yokohama 230-0045, Japan. E-mail: akiko.minoda@riken.jp; Tel: 81-45-503-9309.

Ming Ta Michael Lee, Genomic Medicine Institute, Geisinger Health System, 100 N. Academy Ave. Danville, PA, 17822, USA. E-mail: mlee2@geisinger.edu; Tel: 1-570-214-1005.

Figure S1

a

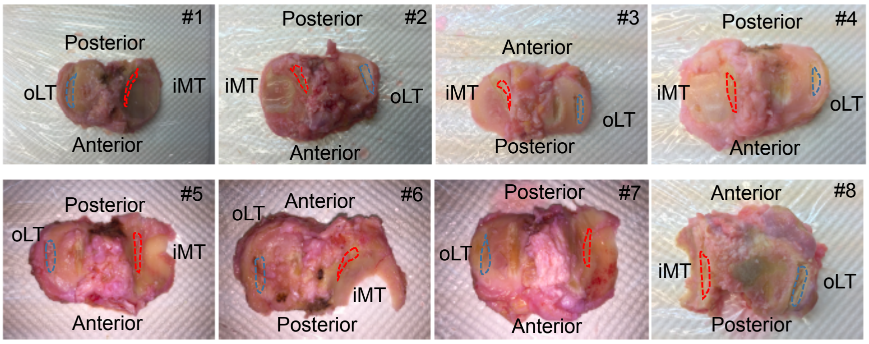

b

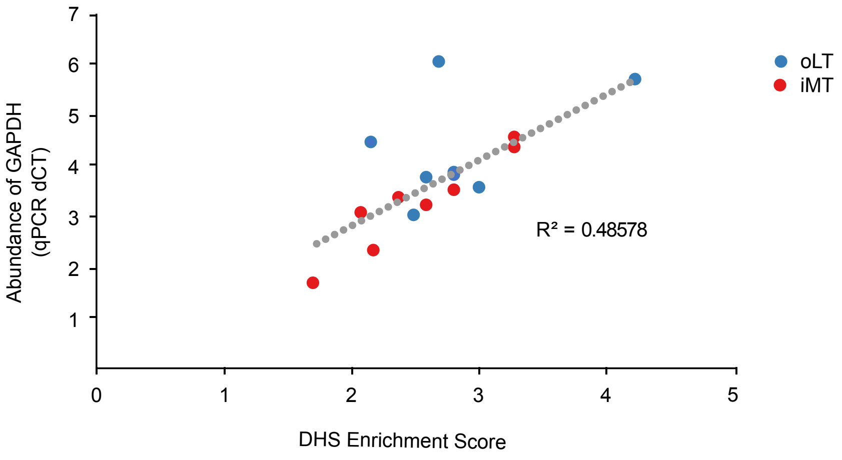

Supplementary Figure S1. ATAC-seq of human cartilage. (a) Images of the dissected cartilage tissue from fresh articular tibial bone for all patients. Section regions of tibial plateau: outer region of lateral tibial plateau (oLT) exhibited macroscopically normal cartilage with a visibly smooth cartilage surface, and inner region of medial tibial plateau (iMT) had visible severe erosion of cartilage. (b) Enrichment qPCR of a housekeeping gene (*GAPDH*) over a heterochromatin region is correlated with enrichment of ATAC-seq signal in DHS regions.

Figure S2

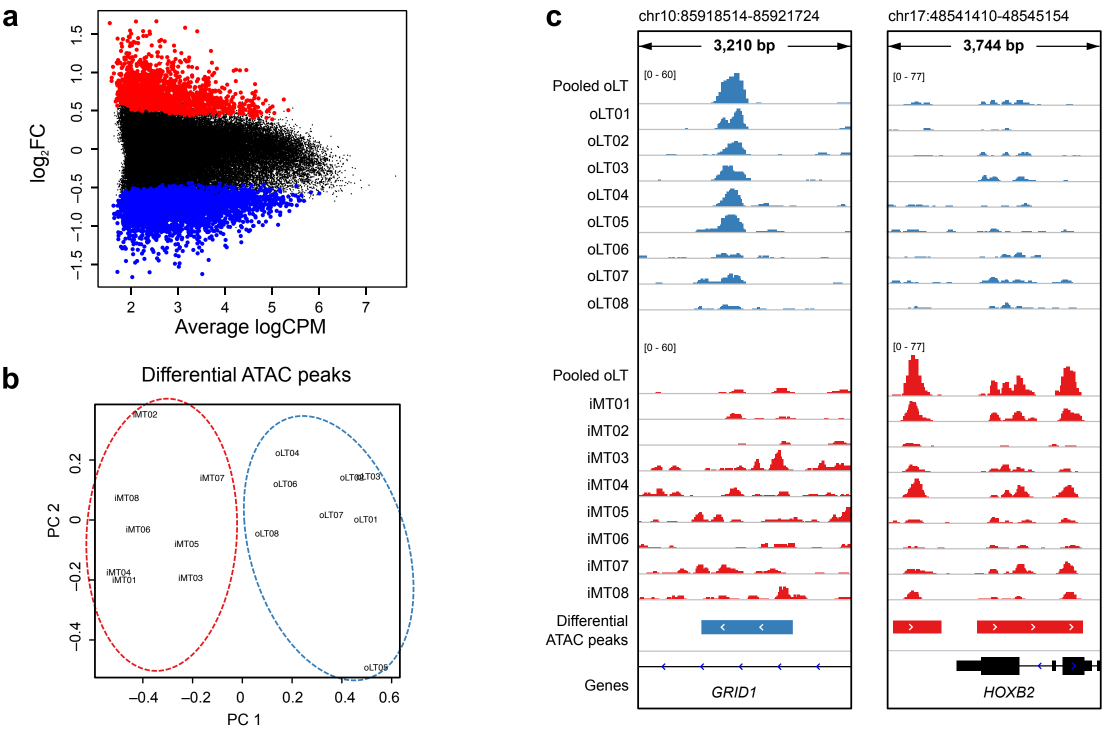

Supplementary Figure S2. Differential ATAC-seq peaks. (a) Mean-difference plot of all ATAC-seq peaks. Red and blue dots represent peaks that are more- and less-accessible in iMT, respectively. (b) Principal Component Analysis of differential ATAC peaks between oLT (blue) and iMT (red). (c) Genome browser views showing consistency of ATAC-seq signals across patients at example loci.

Figure S3

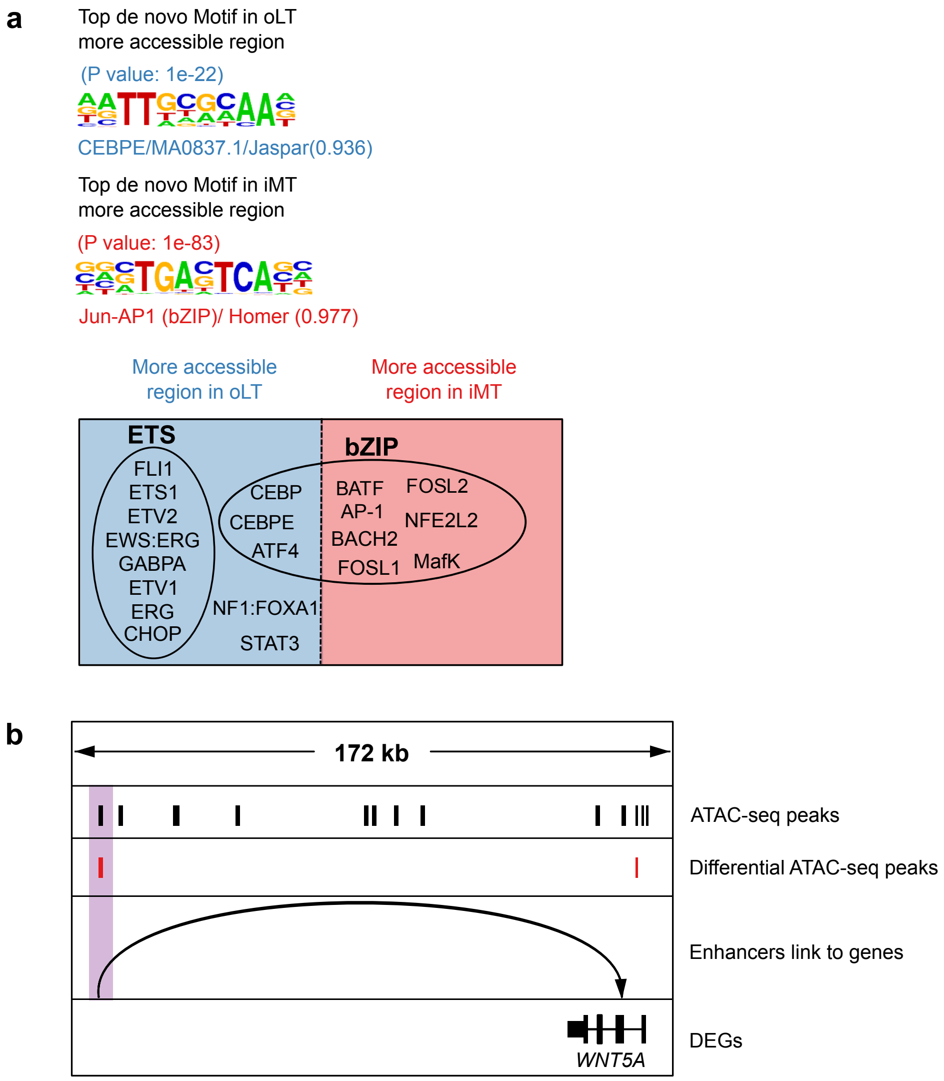

Supplementary Figure S3. Data integration. (a) Top *de novo* motif (top) and top predicted known transcription factors (bottom) enriched in differentially accessible regions. (b) An example gene (*WNT5A*) that is dysregulated at all three criteria (promoter accessibility, enhancer accessibility and expression).
